# Anti-Indigenous bias of medical school applicants: a cross-sectional study

**DOI:** 10.1186/s12909-022-03739-3

**Published:** 2022-09-19

**Authors:** Pamela Roach, Santanna Hernandez, Amanda Carbert, Rabiya Jalil, Remo Panaccione, Shannon M. Ruzycki

**Affiliations:** 1 Department of Family Medicine, Cumming School of Medicine, University of Calgary, Calgary, AB Canada; 2 Cumming School of Medicine, University of Calgary, Calgary, AB Canada; 3 Department of Medicine, Cumming School of Medicine, University of Calgary, Calgary, AB Canada; 4 Department of Community Health Sciences, Cumming School of Medicine, University of Calgary, Calgary, AB Canada; 5 Calgary, Canada

**Keywords:** Racism, Anti-indigenous bias, Discrimination, Medical school application

## Abstract

**Background:**

Structural and interpersonal anti-Indigenous racism is prevalent in Canadian healthcare. The Truth and Reconciliation Commission calls on medical schools to address anti-Indigenous bias in students. We measured the prevalence of interpersonal anti-Indigenous bias among medical school applicants to understand how the medical school selection process selects for or against students with high levels of bias.

**Methods:**

All applicants to a single university in the 2020–2021 admissions cycle were invited to participate. Explicit anti-Indigenous bias was measured using two sliding scale thermometers. The first asked how participants felt about Indigenous people (from 0, indicating ‘cold/unfavourable’ to 100, indicating ‘warm/favourable’) and the second asked whether participants preferred white (scored 100) or Indigenous people (scored 0). Participants then completed an implicit association test examining preferences for European or Indigenous faces (negative time latencies suggest preference for European faces). Explicit and implicit anti-Indigenous biases were compared by applicant demographics (including gender and racial identity), application status (offered an interview, offered admission, accepted a position), and compared to undergraduate medical and mathematics students.

**Results:**

There were 595 applicant respondents (32.4% response rate, 64.2% cisgender women, 55.3% white). Applicants felt warmly toward Indigenous people (median 96 (IQR 80–100)), had no explicit preference for white or Indigenous people (median 50 (IQR 37–55), and had mild implicit preference for European faces (− 0.22 ms (IQR -0.54, 0.08 ms)). There were demographic differences associated with measures of explicit and implicit bias. Applicants who were offered admission had warmer feelings toward Indigenous people and greater preference for Indigenous people compared to those were not successful.

**Conclusions:**

Medical school applicants did not have strong interpersonal explicit and implicit anti-Indigenous biases. Outlier participants with strong biases were not offered interviews or admission to medical school.

**Supplementary Information:**

The online version contains supplementary material available at 10.1186/s12909-022-03739-3.

## Background

Addressing racism, colonialism, and oppression in medical education is an urgent priority [1]. The Jones’ framework [2] demonstrates that this discrimination may be interpersonal [3, 4] or structural [5–7]. Structural and interpersonal racism are determinants of health [8], and are known to harm patients [9, 10], trainees [4], and faculty physicians (Roach P, Ruzycki SM, Hernandez S, Charbertt A, Holroyd-Leduc J, Ahmed S, et al: Explicit and implicit anti-Indigenous bias among Albertan physicians: a cross-sectional study, submitted). The Truth and Reconciliation Commission’s (TRC) health-related Calls to Action requires Canadian healthcare institutions to examine their contributions to ongoing oppression of Indigenous people [11]. In response, the Association of Faculties of Medicine of Canada (AFMC) has released a roadmap for concrete institutional change to address structural racism and fulfill their social accountability mandates with respect to Indigenous health [12]. All Canadian medical schools have committed to this roadmap, beginning with ‘addressing anti-Indigenous sentiment within the medical school’. [12] As such, our school of medicine and its embedded Indigenous Health Program’s mission is to ‘confront issues faced by Indigenous people in the healthcare system and in our (medical school)’. [13] Interpersonal anti-Indigenous sentiments may be explicit, referring to expressed beliefs based on stereotypes that may be harmful, or implicit, the unconscious beliefs based on a society’s values and culture [14].

Operationally, addressing anti-Indigenous sentiments requires measurement of these beliefs among those applying to and accepted to undergraduate medical education programs in Canada. Together, these objectives informed a need to examine the interpersonal bias of applicants and matriculating students to the undergraduate medical program in school of medicine, with an aim of identifying how the application process selects for (or against) individuals with anti-Indigenous attitudes.

## Methods

### Study design

This cross-sectional survey measured demographics and explicit and implicit anti-Indigenous bias. Participants were informed that the purpose of the study was to better understand the demographics of medical school applicants and their feelings toward Indigenous people in Canada; once data collection was complete, participants were informed via e-mail that the responses would be used to measure explicit and implicit anti-Indigenous bias. All participants provided informed consent. Participation was voluntary and not compensated. Results of the study were not used to inform admission decisions. Data was analyzed following complete matriculation of the Class of 2024. This study was approved by our institutional ethics review board (July 21, 2020; REB20–0077).

### Setting & sampling

Applicants to the undergraduate medical education program at the Cumming School of Medicine (University of Calgary) in the 2020–2021 application cycle were approached for participation after submitting their application (*n* = 1837). Applicants must be Canadian citizens, permanent residents, or refugees. Applicants submit an electronic package (‘file’) that contains reference letters, a personal statement, a resume of work and volunteer activities, Medical College Admissions Test (MCAT) score, and undergraduate/postgraduate education transcripts [15]. This file is scored by two reviewers using a standard rubric. A proportion of applicants with the highest scores are invited to a multiple-mini-interview, where the applicant is interviewed by faculty, medical students, and general public stakeholders using a variety of scenarios and topics. The final applicant score is comprised of 50% from the interview and 50% from the file review. The top ranked applicants are offered a position in the matriculating class and an additional number of candidates who meet admissions criteria are placed on the waitlist. While racist and other discriminatory attitudes are not specifically elicited in the evaluation of applicants, these attitudes are named as incompatible with admission to the Cumming School of Medicine and result in termination of the application regardless of the strength of other aspects of the candidate [15].

Participants were stratified by application status: (1) those who were offered an interview (*n* = 884), (2) those who were declined admission after interview (*n* = 622), (3) those who were waitlisted (*n* = 93), (4) those who were offered admission, including those who declined their position (*n* = 264), and (5) those who accepted the offer of a position in the Class of 2024 (‘matriculants’; *n* = 134).

Two comparator populations were chosen; current medical students in their first, second or third years of medical school (the Classes of 2021, 2022, and 2023, respectively) were invited by posting on the class electronic message board and a single e-mail invitation using the class listserve (*n* = 487), and undergraduate students in the Faculty of Mathematics at our same university were invited by a single e-mail to all students enrolled in a senior level course restricted to math majors from their Department Head (*n* = 160). These comparator groups were selected because we were not sure if the demographics and attitudes of medical school applicants would be more like current medical students or other undergraduate STEM students. Undergraduate math majors were considered less likely than other science majors to be applying to medical school [16] and therefore would have less overlap in participant populations than other undergraduate science degrees.

### Outcomes measures

The survey was developed and pilot-tested by a diverse group of medical students and faculty members, including multiple Indigenous study team members (Additional file 1: Appendix 1). There were 8 demographic questions aimed at describing diversity based on protected characteristics in the Canadian Charter of Rights and Freedoms (e.g., gender, sexual orientation, ability, race, ethnicity, Indigenous status). Questions were developed using best practices for sensitive survey development. Multiple responses were permitted for most questions.

Explicit anti-Indigenous bias was measured using a sliding thermometer approach [17, 18] with two questions. The first asked participants to indicate their feelings towards Indigenous people by sliding an arrow from “Cold/Unfavourable” (scored as 0) to “Warm/Favourable” (scored as 100), with a neutral option (scored as 50). The second asked participants to indicate whether they preferred “white people” (scored as 100), “Indigenous people” (scored as 0) or had “No preference” (scored as 50) by sliding an arrow.

Implicit anti-Indigenous bias was assessed using an implicit association test (IAT) designed by researchers at the University of Toronto and Well Living House in Toronto, Ontario, Canada [19]. The Indigenous IAT asked participants to match words with positive or negative connotations with the faces of European or Indigenous people, in random order. The time latency (measured in milliseconds) between matching positive and negative words with European faces and positive and negative words with Indigenous faces is used to estimate an implicit association favouring European or Indigenous faces. Negative latencies suggest a preference for European faces and positive latencies suggest a preference for Indigenous faces. Latencies greater than + 0.65 ms are considered strong preferences for Indigenous faces, within + 0.35 to + 0.64 ms are considered moderate, and + 0.15 to + 0.34 ms + are considered mild preferences. Similarly, less than − 0.65 ms suggest strong preferences for European faces, between − 0.35 to − 0.64 ms are moderate, and − 0.15 to − 0.34 ms are considered mild preferences. Latencies between − 0.15 and + 0.15 ms are considered neutral. The association between IAT and biased behaviours in healthcare is controversial [14], but IATs may be helpful when studying populations rather than individuals [20] and as a learning and reflection tool rather than as a diagnostic tool [21].

Based on feedback from Indigenous study team members that reading and responding to questions about anti-Indigenous racism may be traumatizing for applicants who identify as Indigenous [22], participants who self-identified as Indigenous did not complete the explicit and implicit bias measurement questions.

### Data analysis

Descriptive statistics are reported for demographic characteristics. Explicit and implicit bias measures were non-parametric, and therefore bias was compared between demographic groups using Kruskal-Wallis test or Wilcoxon rank-sum test (for multiple group and two-group comparisons, respectively). Only demographic categories with more than 15 participants were compared statistically. To protect participant identity for those in smaller demographic groups during analysis, race was categorized as white or from a racially marginalized group (“BIPOC”, referring to Black, Indigenous, and People of Colour, a heterogenous group of people who are marginalized due to their appearance) and those with non-binary gender, a gender that was not listed, who were gender diverse or Two Spirit were combined into the group “non-binary gender”.

All participants who answered at least one survey question were included. To assess missingness and nonresponse bias, we compared the demographics of participants to the known demographics of the target population, when available [23]. The Cumming School of Medicine collects only the sex (female, male, or X) [24] and Black or Indigenous race/ethnicity/ancestry among applicants. For this reason, our ability to compare the study participant demographics to what is known about the demographics of non-participants is limited for some characteristics. The correlation between the explicit and implicit measures is shown in eFigure 1 and 2. Data analysis was performed in Stata (version 14; College Station TX).

## Results

There were 988 medical school applicants who opened the survey (open rate 53.8%) and 595 who completed at least one question (response rate 32.4%). There were 25 undergraduate math student and 66 current medical student respondents (15.6 and 13.6% response rate, respectively). Most applicants identified as cisgender women (*n* = 382, 64.2%), white race (*n* = 329, 55.3%), straight (*n* = 487, 82.7%) and having no disability (*n* = 407, 68.4%) (Table 1). Over one third of applicant respondents identified as a visible minority (*n* = 209, 35.5%) and less than one fifth identified as a non-visible minority (*n* = 105, 18.1%). Cisgender men and Black applicants were overrepresented and Indigenous applicants were underrepresented in our sample compared to data collected on all applicants by the medical school (eTable 1).

**Table 1:** Demographic characteristics of all medical school applicant participants, matriculating participants, undergraduate math student respondents and current medical student respondents at the University of Calgary

	Applicant Participants n (%)	Matriculant Participants (Class of 2024) n (%)	Math Students n (%)	Current Medical Student Participants n (%)^b^
Total number	1834	134	160	487
Participants (Response rate)	595^a^ (32.4)	37 (27.6)	25 (15.6)	66 (13.6)
Gender				
Cisgender women	382 (64.2)	21 (60.0)	11 (45.8)	46 (83.6)
Cisgender men	199 (33.5)	12 (34.3)	11 (45.8)	6 (10.9)
A non-binary gender^c^	13 (2.2)	2 (5.8)	2 (8.3)	3 (5.4)
Age				
< 20 years	29 (5.0)	3 (9.1)	17 (68.0)	0
21–25 years	376 (64.8)	16 (48.5)	5 (20.0)	30 (54.6)
26–30 years	93 (16.0)	6 (18.2)	0	20 (36.4)
31–35 years	56 (9.7)	8 (24.2)	2 (8.0)	5 (9.1)
36–40 years	11 (1.9)	0	1 (4.0)	0
41–45 years	10 (1.7)	0	0	0
46–50 years	4 (0.7)	0	0	0
> 55 years	1 (0.2)	0	0	0
Race				
Black	42 (7.1)	5 (13.5)	1 (4.0)	2 (3.0)
White	329 (55.3)	17 (48.6)	8 (32.0)	32 (48.5)
Indigenous	19 (3.2)	1 (2.9)	2 (8.0)	3 (4.5)
Middle Eastern	38 (6.4)	4 (11.4)	2 (8.0)	5 (7.6)
Asian	168 (28.2)	9 (25.7)	10 (40.0)	15 (22.7)
Hispanic/Latinx	11 (1.8)	1 (2.9)	2 (8.0)	0
LGBTQ2S+ Community				
Yes	76 (12.9)	5 (14.7)	2 (8.3)	14 (25.5)
No	487 (82.7)	28 (82.4)	20 (83.3)	41 (74.6)
Abilities				
Other Ability	188 (31.6)	15 (40.5)	0	29 (43.9)
No Disability	407 (68.4)	22 (64.7)	13 (52.0)	37 (56.1)
Minority				
Visible Minority	209 (35.5)	12 (35.3)	8 (33.3)	22 (40.0)
Non-visible Minority	105 (18.1)	12 (35.3)	6 (25.0)	14 (26.4)

All identities are self-reported. Multiple selections were allowed

*LGBTQ2S+* Lesbian, gay, bisexual, transgender, queer, and other sexual orientation community members

Other ability = any sensory, learning, mobility, chronic illness, mental health, temporary illness, or other impairment

^a^ 988 applicants opened the survey link (53.8%) and 595 completed at least one question

^b^ Current medical student participants were those enrolled in their first (Class of 2023), second (Classof 2022) or third (Class of 2021) year of medical school

^c^ Two Spirit, gender diverse, non-binary, transgender, and people whose gender was not listed were combined into the group “non-binary gender” to prevent identifiability due to smaller numbers across individual groups

Explicit and implicit anti-Indigenous bias measures, stratified by participant demographics, are shown in Table 2. Cisgender men had greater explicit anti-Indigenous bias than cisgender women (median feeling of warmth, 90 (IQR 75–100) versus 100 (IQR 83–100), respectively; *p* = 0.001, z = 3.30, *r* = 0.14) though both groups had a similar neutral preference for white and Indigenous people (median preference, 50 (IQR 46.5–58) versus 50 (IQR 39–55), respectively; *p* = 0.41, z = − 0.825, *r* = 0.03). Conversely, BIPOC participants had stronger preference for Indigenous people compared to white participants, whose preferences were generally neutral (median preference 49 (IQR 33–52) versus 50 (49–56.5); *p* = 0.02, z = 2.85, *r* = 0.12). Cisgender men respondents also had stronger implicit anti-Indigenous associations than cisgender women (median latency − 0.31 ms (IQR − 0.62-0.00 ms) versus − 0.19 ms (IQR -0.47 − + 0.15 ms); *p* < 0.001 z = 3.22, *r* = 0.13). Similarly, white participants also had stronger implicit anti-Indigenous association than BIPOC participants (− 0.31 ms (IQR -0.61 − + 0.01 ms) versus − 0.18 ms (IQR -0.36 − + 0.15 ms); *p* < 0.0001 z = − 3.55, *r* = 0.14).

**Table 2:** Explicit and implicit anti-Indigenous measures of medical school applicant respondents, stratified by demographic data

	How do you feel about Indigenous people?^**a**^ (median, IQR)	Do you prefer white people or Indigenous people?^**b**^ (median, IQR)	IAT (median, IQR)
Gender			
Cisgender women	100 (83–100)	50 (39–55)	− 0.19 (− 0.47, 0.15)
Cisgender men	90 (75–100)	50 (46.5–58)	− 0.31 (− 0.62, 0.00)
A non-binary gender^c^	100 (73–100)	31 (25–42)	0.10 (− 0.18, 1.00)
Wilcoxon rank-sum test^d^	*p* = 0.001	*p* = 0.44	*p* = 0.0005
Age			
< 20 years	97.5 (80–100)	47 (37–54)	− 0.20 (− 0.31, 0.13)
21–25 years	96 (79–100)	50 (45.5–55)	− 0.24 (− 0.57, 0.06)
26–30 years	87 (79–100)	50 (32–55)	− 0.20 (− 0.52, 0.15)
31–35 years	100 (90–100)	50 (36–55)	− 0.17 (− 0.59, 0.21)
36–40 years	100 (100–100)	27 (0–47)	0.01 (− 0.50, 0.30)
41–45 years	100 (88–100)	58 (58–58)	0.41 (− 0.76, 0.29)
46–50 years	93 (85.5–100)	–	− 0.63 (− 0.63, − 0.63)
> 55 years	88 (88–88)	–	− 0.19 (− 0.19, − 0.19)
Kruskal-Wallis test	*p* = 0.01	*p* = 0.33	*p* = 0.69
Race			
Black	100 (83–100)	47 (33–50)	− 0.29 (− 0.50, 0.25)
White	98 (82–100)	50 (49–56.5)	− 0.31 (− 0.61, 0.01)
Indigenous	–	–	–
Middle Eastern	100 (95.5–100)	38 (0–47)	− 0.28 (− 0.43, 0.13)
Asian	93.5 (77.5–100)	50 (37–61)	− 0.18 (− 0.34, 0.10)
Hispanic/Latinx	93.5 (87–100)	51.5 (51–52)	0.04 (− 0.42, 0.36)
Wilcoxon rank-sum test^e^	*p* = 0.42	*p* = 0.02	*p* = 0.0004
LGBTQ2S+ Community			
Yes	100 (82–100)	48 (33–56)	−0.26 (− 0.60, 0.11)
Unsure	98 (85–100)	47 (44–54)	− 0.07 (− 0.30, 0.21)
No	96 (80–100)	50 (41–55)	− 0.22 (− 0.54, 0.10)
Wilcoxon rank-sum test^f^	*p* = 0.29	*p* = 0.17	*p* = 0.11
Minority			
Visible Minority	98 (80–100)	48 (33–56)	−0.26 (− 0.60, 0.11)
Non-visible Minority	100 (84–100)	43 (28–52)	−0.23 (− 0.54, 0.08)

Statistical comparison was only performed when groups had more than 15 respondents

*IAT* implicit association test, *LGBTQ+* Lesbian, gay, bisexual, transgender, queer, and other sexual orientation community members

Other ability = any sensory, learning, mobility, chronic illness, mental health, temporary illness, or other impairment

^a^ Responses used a sliding thermometer from 0 to 100, where 100 is labelled “Warm/Favourable” and 0 is labelled “Cold/Unfavourable” and 50 indicates neutral

^b^ Responses used a sliding thermometer from 0 to 100, where 100 is labelled “white people”, 0 is labelled “Indigenous people” and 50 is labelled “no preference”

^c^ Two Spirit, gender diverse, non-binary, transgender, and people whose gender was not listed were combined into the group “non-binary gender” to prevent identifiability due to smaller numbers across individual groups

^d^ Comparison between cisgender women and cisgender men

^e^ Comparison between white and Black, Indigenous and People of Colour participants

^f^ Comparison between “Yes and Unsure” members of the LGTBQ+ community to “No” respondents

**Fig. 1:**
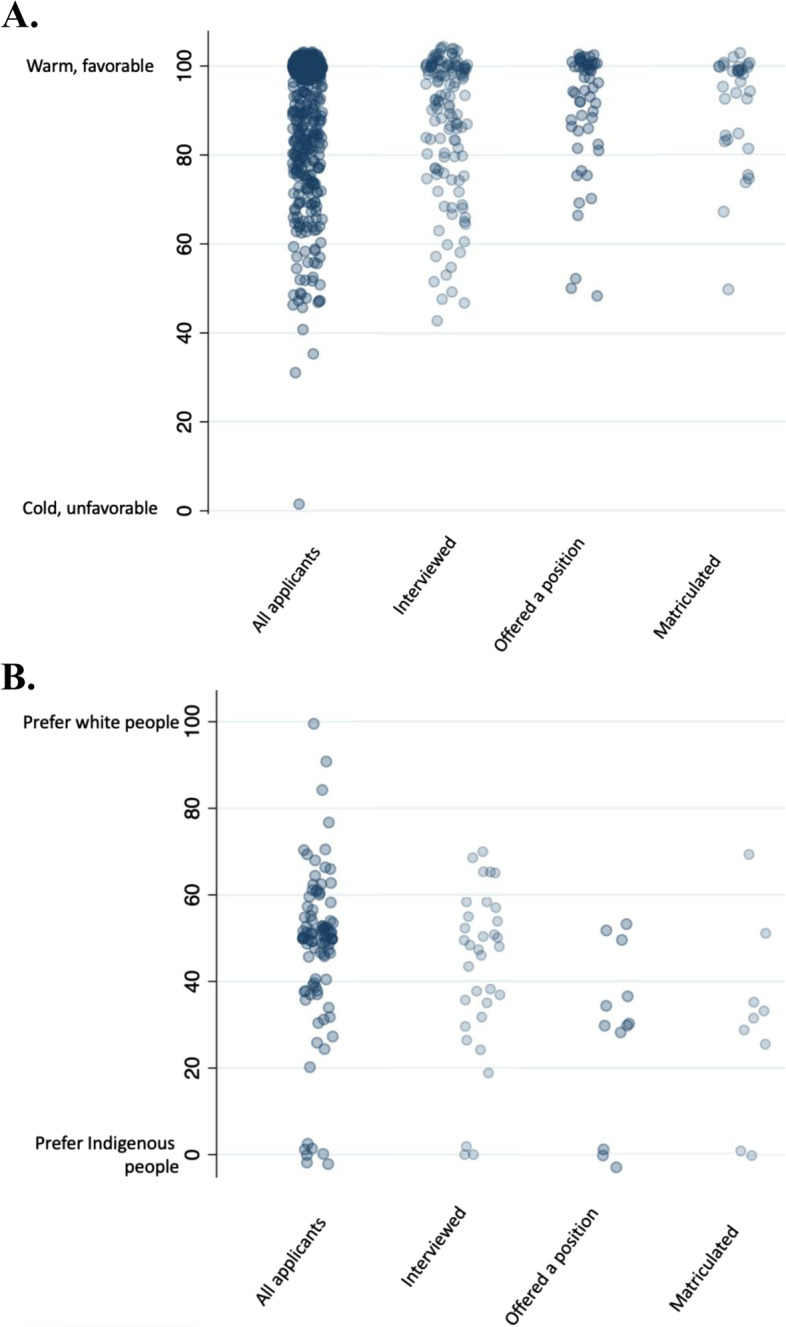
Individual medical school applicant participant responses to explicit anti-Indigenous bias questions (blue circles), stratified by application status. **A** How do you feel toward Indigenous people? **B** Do you prefer white people or Indigenous people?

**Fig. 2:**
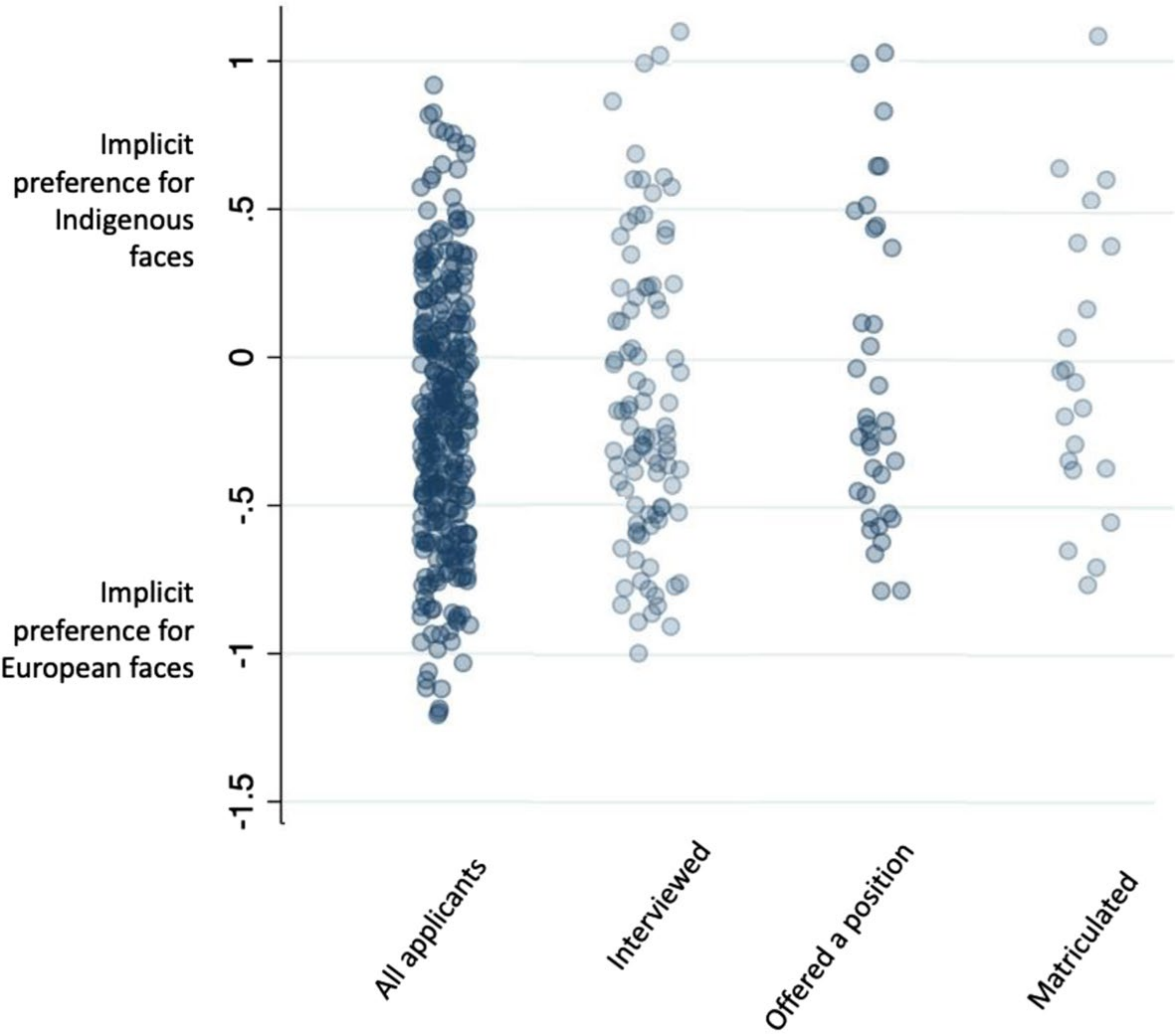
Individual medical school applicant participant responses scores (blue circles) on the anti-Indigenous implicit association test, stratified by application status

Measures of explicit and implicit bias, stratified by application status, are shown in Table 3 and Figs. 1 and 2. Overall, medical school applicant participants felt warmly toward Indigenous people (median 96; IQR 80–100), had no explicit preference for white or Indigenous people (median 50, IQR 37–55), and had mild implicit preference for European faces (median latency − 0.22 ms; IQR -0.54 − + 0.08 ms). Undergraduate math students had greater implicit and explicit anti-Indigenous bias compared to medical school applicants (Table 3). There was no difference in the explicit and implicit bias measures between medical school applicant participants who were and were not offered an interview (Figs. 1 and 2); however, applicants who were offered admission and who matriculated in the Class of 2024 had a median explicit bias score that suggested a preference for Indigenous people over white people compared to participants who were not offered admission (Table 3). Similarly, participants who matriculated in the Class of 2024 had a median implicit association that indicated no preference for European or Indigenous faces compared to those who were not admitted.

**Table 3:** Measures of explicit and implicit anti-Indigenous bias of medical school applicants, stratified by application status, compared with current medical students and undergraduate math students

	How do you feel about Indigenous people?^**a**^ (median, IQR)	Do you prefer white people or Indigenous people?^**b**^ (median, IQR)	IAT^**c**^ (ms) (median, IQR)
Applicants	96 (80–100)	50 (37–55)	−0.22 (− 0.54, 0.08)
Math Students	77 (58–100)	57.5 (52.5–64.5)	− 0.30 (− 0.74, 0.07)
Current Medical Students	93 (77–100)	51 (30–71)	−0.16 (− 0.41, 0.18)
Kruskal-Wallis test	*p* = 0.05	*p* = 0.11	*p* = 0.44
Not interviewed	98 (80–100)	50 (40–54)	−0.22 (− 0.52, 0.07)
Interviewed	94 (79–100)	50 (32–55)	−0.25 (− 0.55, 0.13)
Wilcoxon rank-sum test	*p* = 0.52	*p* = 0.43	*p* = 0.65
Rejected	90 (75–100)	51 (48–58)	−0.31 (− 0.63, 0.11)
Waitlisted	90 (75–100)	50 (43.5–58.5)	−0.11 (− 0.26, 0.13)
Accepted	96.5 (86–100)	31 (13.5–43)	− 0.27 (− 0.49, 0.34)
Wilcoxon rank-sum test	*p* = 0.47	*p* = 0.02	*p* = 0.43
Not admitted	96 (79–100)	50 (40–55)	−0.23 (− 0.55, 0.08)
Matriculated	100 (86–100)	32 (27–35)	−0.07 (− 0.32, 0.37)
Wilcoxon rank-sum test	*p* = 0.36	*p* = 0.01	*p* = 0.12

*IAT* Anti-Indigenous Implicit Association Test, *IQR* interquartile range

^a^ Responses used a sliding thermometer from 0 to 100, where 100 is labelled “Warm/Favourable” and 0 is labelled “Cold/Unfavourable” and 50 indicates neutral

^b^ Responses used a sliding thermometer from 0 to 100, where 100 is labelled “white people”, 0 is labelled “Indigenous people” and 50 is labelled “no preference”

^c^ Scored in time latencies, where negative results suggest a preference for European faces and positive scores suggest a preference for Indigenous faces. Scores greater than |0.65 ms| suggest strong preferences, between |0.64 to 0.35 ms| suggest moderate preferences, between |0.15 to 0.34 ms| suggest mild preferences, and scores between |0.15 and 0 ms| suggest no preference

## Discussion

This study aimed to characterize interpersonal anti-Indigenous bias among medical school applicants, whether this bias differed between applicants to medical school and other undergraduate students, and if bias was associated with success during the application process. We found that successful applicants had less implicit and explicit anti-Indigenous bias compared to unsuccessful applicants and other undergraduate science students. Outlier applicants with strong explicit and implicit anti-Indigenous biases were removed from consideration for admission after file review and interviews. It is not clear whether this was due to chance, the identification of this bias during selection leading to disqualification, or another factor that is associated with lower application scores and anti-Indigenous bias. More study is required to determine whether the current processes are effective in identifying candidates with discriminatory attitudes, such as racism, that are incompatible with working as a physician.

This study represents the first effort to characterize implicit and explicit interpersonal anti-Indigenous bias among Canadian medical students and medical school applicants. Medical students have been demonstrated to have biases against several protected groups, including sexual and gender minorities, racially marginalized people, and people with intellectual disabilities [2, 7, 11–15, 18–23]. These biases are often implicit, but explicit discriminatory beliefs have been endorsed by medical students in several studies [25, 26]. In our study, medical school applicants had lower median explicit and implicit anti-Indigenous measures than other undergraduate students. This result is reassuring, given that medical school applicants are a select group with higher socioeconomic status and most have greater privilege than the general population [27]. Further, applicants, matriculating students, and current medical students all felt more warmly toward Indigenous people than current practicing physicians (Roach P, Ruzycki SM, Hernandez S, Charbertt A, Holroyd-Leduc J, Ahmed S, et al: Explicit and implicit anti-Indigenous bias among Albertan physicians: a cross-sectional study, submitted) and had less implicit preference for European faces. It is not known how these biases change throughout medical training; while some studies have shown reduced bias with exposure to marginalized groups in medical training [28–30], other studies have demonstrated increasing bias with exposure to negative role modelling by senior physicians [31–34]. Therefore, it will be important to both monitor bias over time in medical trainees and to work to correct existing systemic anti-Indigenous bias in medical culture.

The applicants who had the greatest individual bias measures were not admitted to our medical school. Due to small numbers, it is not clear whether this is attributable to a component of our admissions process rather than chance alone. Monitoring this effect over time may inform whether additional processes are required to identify individuals with racist attitudes during the application process. Though the results of explicit bias measures collected for this study were not used to inform admissions decisions, it may be reasonable to incorporate these questions in the applicant file or interview to ensure that applicants with explicit biases are not admitted to medical school. Due to the limitations of implicit association tests and contradicting evidence linking implicit association scores to behaviours, implicit bias measures should not be included in medical school admissions processes.

It is likely that social desirability bias influenced our results, in particular for medical applicants who are entering a high-stakes, competitive process and would be motivated to portray themselves as positively as possible. Medical school applicants may have altered their responses, particularly to explicit anti-Indigenous bias measures, to appear more suitable for medical school. For this reason, it is likely that our results underestimate explicit anti-Indigenous bias among respondents. In addition, our response rate of about 30% and proportion of missed responses limits our ability to precisely estimate the diversity of the respondents. Given that 988 participants opened the survey but only 595 completed any questions, it is likely the topics deterred many participants. This nonresponse bias is very likely to be non-random and unpredictable, and its influence on our findings is unknown. Despite this limitation, these data represent the most comprehensive enumeration of the bias of medical school applicants. Lastly, the association between implicit association measures and behaviours is not conclusive. Implicit bias measures should be considered exploratory only.

## Conclusions

This cross-sectional survey, while limited by response rate, suggests that medical school applicants and matriculants have less explicit and implicit anti-Indigenous bias than other undergraduate students. Further data is needed to understand how bias changes throughout medical training.

## Supplementary Information

**Additional file 1: Appendix 1.** Survey instrument. **Appendix 2. eTable 1**. Comparison of participant demographics with the target population demographics. **Appendix 3. eFigure 1**. Correlation of explicit anti-Indigenous bias measures with implicit anti-Indigenous bias. **Appendix 4. eFigure 2**. Correlation between explicit anti-Indigenous bias measures.

## Data Availability

The datasets generated during this current study are not publicly available due to privacy concerns and identifiability of participants, but select data are available from the corresponding author upon reasonable request. The Indigenous Implicit Association Test is available from researchers at Well Living House in Toronto, Canada.
