# Supplementary material for: Anti-Indigenous bias of medical school applicants: a cross-sectional study
Source: BMC Med Educ. 2022 Sep 19;22:683. doi: 10.1186/s12909-022-03739-3 (PMC9484063; doi:10.1186/s12909-022-03739-3)
Supplement: Supplementary file 1 — Additional file 1: Appendix 1. Survey instrument. Appendix 2. eTable 1. Comparison of participant demographics with the target population demographics. Appendix 3. eFigure 1. Correlation of explicit anti-Indigenous bias measures with implicit anti-Indigenous bias. Appendix 4. eFigure 2. Correlation between explicit anti-Indigenous bias measures. [file 12909_2022_3739_MOESM1_ESM.docx]

**Appendices**

Anti-Indigenous Bias of Medical School Applicants by Application Status:

A Cross-Sectional Study

| **Appendix 1.** Survey instrument. | 2 |
| --- | --- |
| **Appendix 2.** eTable 1. Comparison of participant demographics with the target population demographics. | 7 |
| **Appendix 3.** eFigure 1. Correlation of explicit anti-Indigenous bias measures with implicit anti-Indigenous bias. | 8 |
| **Appendix 4.** eFigure 2. Correlation between explicit anti-Indigenous bias measures. | 9 |

Appendix 1. Survey Instrument.

Q1 Please enter your AMCAS number (American Medical College Application Services).

________________________________________________________________

Q2 Do you identify as First Nations, Inuit, or Métis?

- Yes (1)
- No (2)

Skip To: Q15 If Do you identify as First Nations, Inuit, or Métis? = Yes

Q3 How do you feel toward Indigenous people?

|  | Cold, unfavourable | Warm, favourable |
| --- | --- | --- |

| () | 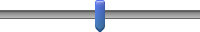 |
| --- | --- |

| Page Break |  |
| --- | --- |

Q4 Please indicate your preference by sliding the pointer.

|  | I prefer Indigenous people | No preference | I prefer white people |
| --- | --- | --- | --- |

|  | 0 | 100 |
| --- | --- | --- |

| () | 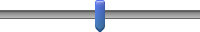 |
| --- | --- |

| Page Break |  |
| --- | --- |

The following information is being collected to understand the characteristics and diversity of people who apply to medical school.

 This information **will not be used** to make admissions decisions.

 This information **will not be accessed by anyone** at the Cumming School of Medicine until July 2021, after the final medical school class has been selected. The data will be linked to the status of your application only (e.g., admitted, offered an interview, not admitted). Your name and other personal information **will not be linked to these data.**

Q5 How old are you?

▼ Under 20 (1) ... Prefer not to answer (10)

Q6 Which of the following most closely represents your gender

- Cisgender man (A doctor identified you as 'male' at birth and you identify as a man now) (1)
- Cisgender woman (A doctor identified you as 'female' at birth and you identify as a woman now) (2)
- Transgender man (A doctor identified you as 'female' at birth and you identify as a man now) (3)
- Transgender woman (A doctor identified you as 'male' at birth and you identify as a woman now) (4)
- Non-binary gender (You identify as another gender besides man and woman) Please describe: (5) ________________________________________________
- Gender diverse (Your gender identity is not well described by conventional genders) Please describe: (6) ________________________________________________
- A gender not listed above. Please describe: (7) ________________________________________________
- Unsure. Please describe: (8) ________________________________________________
- Prefer not to answer (9)

Q7 Which of the following most closely represents your race and/or ethnicity? Please select all that apply.

- Black. Please describe: (1) ________________________________________________
- Caucasian/white (2)
- First Nations (status). Please describe: (3) ________________________________________________
- First Nations (non-status). Please describe: (4) ________________________________________________
- Inuit. Please describe: (5) ________________________________________________
- Métis. (6)
- Middle Eastern. Please describe: (7) ________________________________________________
- Asian. Please describe: (8) ________________________________________________
- Hispanic (9)
- Latinx. Please describe: (10) ________________________________________________
- A race and/or ethnicity that is not listed above: (11) ________________________________________________

Q8 Are you a member of the LGBTQI2S+ community? (Referring to lesbian, gay, bisexual, transgender, queer, intersex, two-spirited, and other types of sexual orientation diversity community?

- Yes. Please describe: (1) ________________________________________________
- Unsure. Please describe: (2) ________________________________________________
- No (3)
- Prefer not to answer. (4)

Q9 How do you define your ability or disability status? We are interested in this identification regardless of whether you typically request accommodations for this disability. Please select all that apply.

- A sensory impairment (e.g., vision or hearing). Please describe: (1) ________________________________________________
- A learning disability (e.g., attention deficit hyperactivity disorder, dyslexia). Please describe: (2) ________________________________________________
- A long-term medical illness (e.g, epilepsy, cystic fibrosis). Please describe: (3) ________________________________________________
- A mobility impairment. Please describe: (4) ________________________________________________
- A mental health disorder. Please describe: (5) ________________________________________________
- A temporary impairment due to illness or injury (e.g., a broken ankle). Please describe: (6) ________________________________________________
- A disability, other ability, or impairment not listed above. Please describe: (7) ________________________________________________
- I do not identify with a disability, other ability, or impairment. (8)
- Prefer not to answer. (9)

Q10 Do you consider yourself a member of a visible minority group? A visible minority group means that someone seeing you for the first time could discriminate against you based on your appearance.

- Yes. Please describe: (1) ________________________________________________
- Maybe. Please describe: (2) ________________________________________________
- No (3)
- Prefer not to answer. (4)

Q11 Do you consider yourself a member of a non-visible minority group? A non-visible minority group means that you may belong to a group that experiences discrimination but a person seeing you for the first time may not easily notice that you belong to this group.

- Yes. Please describe: (1) ________________________________________________
- Maybe. Please describe: (2) ________________________________________________
- No (3)
- Prefer not to answer. (4)

| Page Break |  |
| --- | --- |

**eTable 1.** Comparison of the known demographics of the target population(s) with the participant demographics, by application status.

|  | **All Applicants** | | **Interviewees** | | **Matriculants (Class of 2024)** | |
| --- | --- | --- | --- | --- | --- | --- |
|  | **Study Participants**  n (%) | **CSM Data**  n (%) | **Study Participants**  n (%) | **CSM Data**  n (%) | **Study Participants**  n (%) | **CSM Data**  n (%) |
| Total number (response rate) | 595 (32·4) | 1,834 | 149 (16.8) | 624 | 37 (27·6) | 134 |
| Gender | | | | | | |
| Cisgender women | 382 (64·2) | 1,092 (59.5) | 75 (55.1) | 375 (60.1) | 21 (60·0) | 93 (69.4) |
| Cisgender men | 199 (33·5) | 730 (39.8) | 55 (40.4) | 244 (39.1) | 12 (34·3) | 40 (29.9) |
| A non-binary gender^†^ | 13 (2·2) | 12 (0.7) | 6 (4.4) | 5 (0.8) | 2 (5.8) | 1 (0.7) |
| Age | | | | | | |
| < 20 years | 29 (5.0) | N/A | 6 (4.4) | N/A | 3 (9.1) | N/A |
| 21-25 years | 376 (64.8) |  | 82 (60.3) |  | 16 (48.5) |  |
| 26-30 years | 93 (16.0) |  | 30 (22.1) |  | 6 (18.2) |  |
| 31-35 years | 56 (9.7) |  | 16 (11.8) |  | 8 (24.2) |  |
| 36-40 years | 11 (1.9) |  | 2 (1.5) |  | 0 |  |
| 41-45 years | 10 (1.7) |  | 0 |  | 0 |  |
| 46-50 years | 4 (0.7) |  | 0 |  | 0 |  |
| > 55 years | 1 (0.2) |  | 0 |  | 0 |  |
| Race | | | | | | |
| Black | 42 (7·1) | 73 (4.0) | 10 (7.4) | 33 (5.3) | 5 (13·5) | 10 (7.5) |
| White | 329 (55·3) | N/A | 76 (51.4) | N/A | 17 (48·6) | N/A |
| Indigenous | 19 (3·2) | 44 (2.4) | 7 (4.7) | 43 (6.9) | 1 (2·9) | 9 (6.7) |
| Middle Eastern | 38 (6·4) | N/A | 11 (7.4) | N/A | 4 (11·4) | N/A |
| Asian | 168 (28·2) | N/A | 37 (25.0) | N/A | 9 (25·7) | N/A |
| Hispanic/Latinx | 11 (1·8) | N/A | 2 (1.4) | N/A | 1 (2·9) | N/A |
| LGBTQ2S+ Community | | | | | | |
| Yes | 76 (12·9) | N/A | 27 (20.4) | N/A | 5 (14·7) | N/A |
| No | 487 (82·7) |  | 103 (76.9) |  | 28 (82·4) |  |
| Abilities |  |  |  |  |  |  |
| Other Abilities | 188 (31·6) |  | 59 (39.2) |  | 15 (40·5) |  |
| No Disability | 407 (68·4) |  | 90 (60.8) |  | 22 (64·7) |  |
| Minority |  |  |  |  |  |  |
| Visible Minority | 209 (35·5) |  | 47 (34.8) |  | 12 (35·3) |  |
| Non-visible Minority | 105 (18·1) |  | 28 (20.9) |  | 12 (35·3) |  |

CSM = Cumming School of Medicine, University of Calgary

LGBTQ2S+ = lesbian, gay, bisexual, queer, Two Spirit, referring to community of gender and sex diverse people and people with diverse sexual orientations.

Multiple responses were allowed for most categories.

**eFigure 1.** Correlation between explicit anti-Indigenous bias and implicit anti-Indigenous bias among medical school applicants. (A) How do you feel toward Indigenous people? (B) Do you prefer white people or Indigenous people?


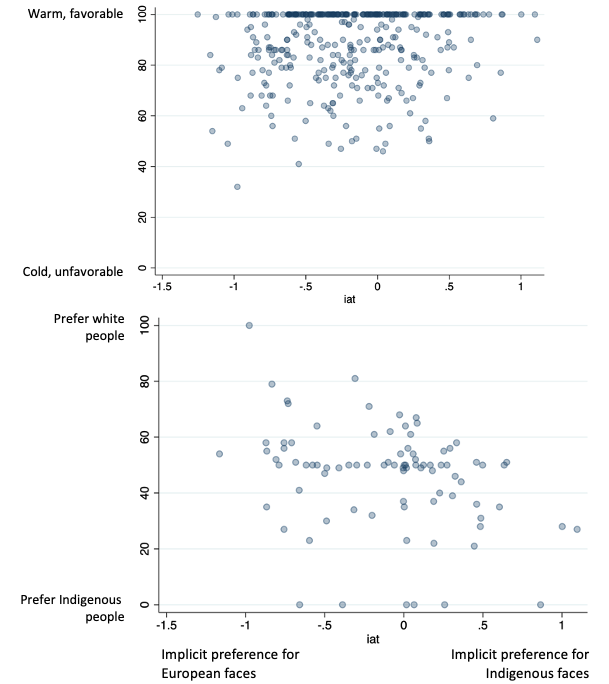


**eFigure 2**. Correlation between explicit anti-Indigenous bias measures among medical school applicants. Vertical axis: Do you prefer white people or Indigenous people? Horizontal axis: How do you feel toward Indigenous people?

**
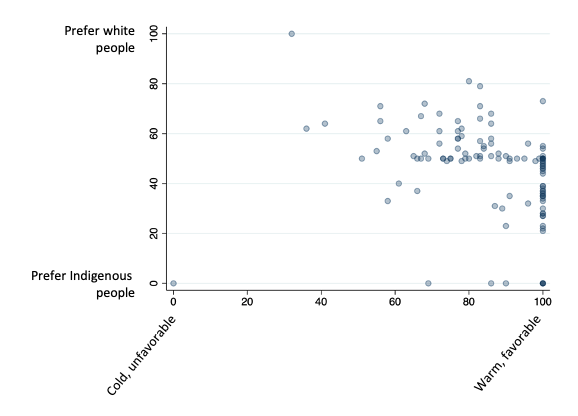
**
